# KLC1-ALK: A Novel Fusion in Lung Cancer Identified Using a Formalin-Fixed Paraffin-Embedded Tissue Only

**DOI:** 10.1371/journal.pone.0031323

**Published:** 2012-02-08

**Authors:** Yuki Togashi, Manabu Soda, Seiji Sakata, Emiko Sugawara, Satoko Hatano, Reimi Asaka, Takashi Nakajima, Hiroyuki Mano, Kengo Takeuchi

**Affiliations:** 1 Pathology Project for Molecular Targets, The Cancer Institute, Japanese Foundation for Cancer Research, Tokyo, Japan; 2 Division of Pathology, The Cancer Institute, Japanese Foundation for Cancer Research, Tokyo, Japan; 3 Division of Functional Genomics, Jichi Medical University, Tochigi, Japan; 4 Department of Comprehensive Pathology, Graduate School, Tokyo Medical and Dental University, Tokyo, Japan; 5 Division of Diagnostic Pathology, Shizuoka Cancer Center, Nagaizumi, Shizuoka, Japan; 6 Department of Medical Genomics, Graduate School of Medicine, University of Tokyo, Tokyo, Japan; The Chinese University of Hong Kong, Hong Kong

## Abstract

The promising results of anaplastic lymphoma kinase (ALK) inhibitors have changed the significance of ALK fusions in several types of cancer. These fusions are no longer mere research targets or diagnostic markers, but they are now directly linked to the therapeutic benefit of patients. However, most available tumor tissues in clinical settings are formalin-fixed and paraffin-embedded (FFPE), and this significantly limits detailed genetic studies in many clinical cases. Although recent technical improvements have allowed the analysis of some known mutations in FFPE tissues, identifying unknown fusion genes by using only FFPE tissues remains difficult. We developed a 5′-rapid amplification of cDNA ends-based system optimized for FFPE tissues and evaluated this system on a lung cancer tissue with *ALK* rearrangement and without the 2 known ALK fusions EML4-ALK and KIF5B-ALK. With this system, we successfully identified a novel ALK fusion, KLC1-ALK. The result was confirmed by reverse transcription-polymerase chain reaction and fluorescence *in situ* hybridization. Then, we synthesized the putative full-length cDNA of *KLC1-ALK* and demonstrated the transforming potential of the fusion kinase with assays using mouse 3T3 cells. To the best of our knowledge, KLC1-ALK is the first novel oncogenic fusion identified using only FFPE tissues. This finding will broaden the potential value of archival FFPE tissues and provide further biological and clinical insights into ALK-positive lung cancer.

## Introduction

Anaplastic lymphoma kinase (ALK) is a receptor tyrosine kinase that was discovered in anaplastic large-cell lymphoma (ALCL) in the form of a fusion protein, NPM-ALK [1], [2]. The formation of a fusion protein with a partner through chromosomal translocations is the most common mechanism of ALK overexpression and ALK kinase domain activation. Recent promising results of clinical trials with an ALK inhibitor, crizotinib, have changed the significance of ALK fusions in lung cancer [3], [4], [5], [6], inflammatory myofibroblastic tumors (IMTs) [7], and ALCL [8]. ALK fusions are no longer mere research targets or diagnostic markers and are now directly linked to the therapeutic benefit of patients.

In lung cancer, 3 fusion partners of ALK have been reported—EML4, TFG, and KIF5B—although the presence of TFG-ALK in lung cancer has not yet been proven with histopathological evidence [9], [10], [11]. In addition to lung cancer, ALK has further been found to generate fusions in ALCL (fused to NPM, TPM3, TPM4, ATIC, TFG, CLTC, MSN, MYH9, or ALO17) [1], [2], [12], [13], [14], [15], [16], [17], [18], [19], IMT (TPM3, TPM4, CLTC, CARS, RANBP2, ATIC, or SEC31A) [19], [20], [21], [22], [23], [24], ALK-positive large B-cell lymphoma (CLTC, NPM, SEC31A, or SQSTM1) [25], [26], [27], [28], and renal cancer (VCL, TPM3 or EML4) (Table 1) [29], [30]. In addition to TFG-ALK in lung cancer, some ALK fusions have been reported without histopathological evidence: TPM4-ALK in esophageal squamous cell carcinoma [31], [32] and EML4-ALK in colon and breast carcinomas [33].

**Table 1:** ALK fusion partners.

Reported year	Partner	Locus	ALK+ALCL	ALK+LBCL	IMT	NSCLC	RCC
1994	NPM	5q35.1	+	+			
1999	TPM3	1p23	+		+		+
1999	TFG	3q12.2	+			+*	
2000	ATIC	2q35	+		+		
2000	TPM4	19p13	+		+		
2001	CLTC	17q23	+	+	+		
2001	MSN	Xp11.1	+				
2002	ALO17	17q25.3	+				
2003	MYH9	22q13.1	+				
2003	RANBP2	2q13			+		
2003	CARS	11p15			+		
2006	SEC31A	4q41		+	+		
2007	EML4	2p21				+	+
2009	KIF5B	10p11.22				+	
2011	SQSTM1	5q35.3		+			
2011	PPFIBP1	12p11			+		
2011	VCL	10q22.2					+
Present study	KLC1	14q32.1				+	

*Histopathological evidence is lacking. Abbreviations: ALCL, anaplastic large cell lymphoma; LBCL, large B-cell lymphoma; IMT, inflammatory myofibroblastic tumor; NSCLC, non-small cell lung carcinoma; RCC, renal cell carcinoma.

Anti-ALK immunohistochemistry played an important role in identifying these ALK fusion partners. Several ALK fusions exhibit a characteristic staining pattern in anti-ALK immunohistochemistry because the subcellular localization of ALK fusion proteins depends on the fusion partner. For example, NPM-ALK, which is the most common fusion in ALK-positive ALCL (85%), exhibits a nuclear and cytoplasmic staining pattern because the heterodimer of NPM and NPM-ALK localizes in the nucleus and the homodimer of NPM-ALK in the cytoplasm; CLTC-ALK exhibits a cytoplasmic granular pattern because it localizes in the small vesicles. If a tumor exhibits an unrecognized anti-ALK staining pattern, the patient may have a novel fusion partner. In addition to the difference in subcellular localization, the difference in staining intensity is a key to identifying novel partners. EML4-ALK is hardly stained by conventional anti-ALK immunohistochemistry [11], [34]. To overcome this limitation, we developed the intercalated antibody-enhanced polymer (iAEP) method, which moderately increases sensitivity in the immunohistochemical detection system, and EML4-ALK was consistently stained with this method [11]. This indicated that a tumor that is positively immunostained for ALK only by a sensitive immunohistochemistry method but not by conventional methods may harbor a novel ALK fusion. Based on this hypothesis, we successfully identified PPFIBP1-ALK in 2 IMT cases that were positive in anti-ALK immunohistochemistry only when stained by the iAEP method [35].

Anti-ALK immunohistochemistry may thus be useful to detect candidate tumors for a novel ALK fusion. However, to identify the fusion partner, other molecular techniques are usually required such as 5′-rapid amplification of cDNA ends (5′-RACE) or inverse reverse-transcription polymerase chain reaction (RT-PCR). To the best of our knowledge, no novel oncogenic fusions have been discovered using formalin-fixed paraffin-embedded (FFPE) tissues only because nucleic acids extracted from FFPE tissues are severely degraded during the fixation process. In the present study, we developed a 5′-RACE method optimized for *ALK* fusion partner detection that was applicable to FFPE tissues and identified a novel fusion, kinesin light chain 1 (KLC1)-ALK, in lung cancer by using only an FFPE tissue.

## Methods

### Materials

**Figure 1:**
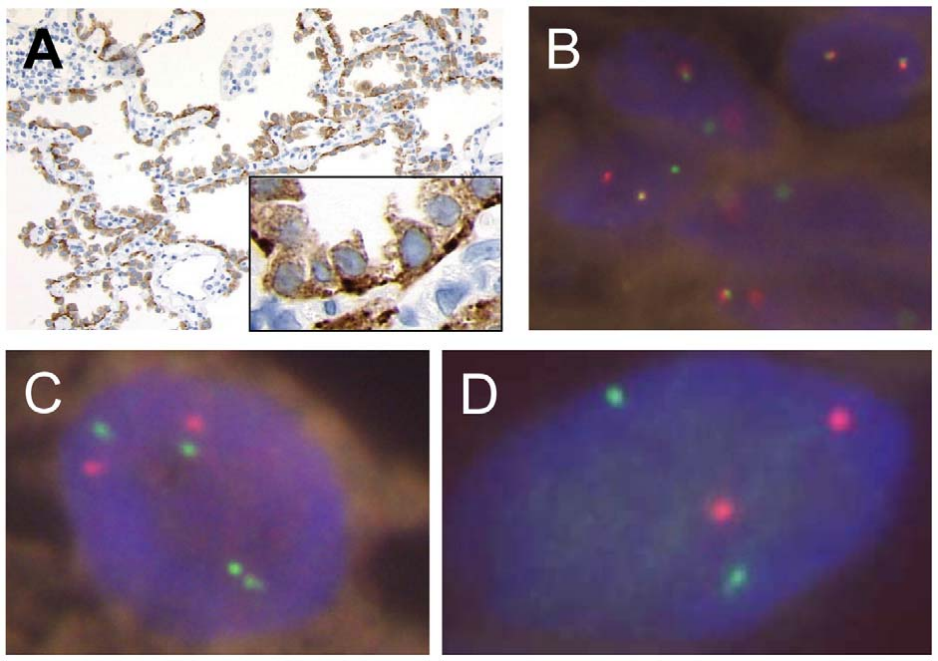
ALK-rearranged lung adenocarcinoma without EML4-ALK and KIF5B-ALK. Panel A shows the results of anti-ALK immunohistochemistry with the iAEP method on pulmonary adenocarcinoma in situ, nonmucinous. The staining pattern was diffusely cytoplasmic. The basal side of tumor cells was more strongly stained, indicating an uneven subcellular localization of KLC1-ALK protein. FISH analyses revealed that this case was positive in the split assay for *ALK* (Panel B: individual 5′- and 3′-signals are observed) and negative in *EML4-ALK* and *KIF5B-ALK* fusion assays (Panel C: *EML4*, red; *ALK*, green; Panel D: *KIF5B*, green; *ALK*, red).

A FFPE tissue block of pulmonary adenocarcinoma in situ, nonmucinous (formerly called bronchioloalveolar carcinoma) [36], which was excised from a 47-year-old female patient was used [37]. This carcinoma was negative for EML4-ALK and KIF5B-ALK, although the presence of *ALK* rearrangement was confirmed by anti-ALK iAEP immunohistochemistry and a split fluorescence in situ hybridization (FISH) assay for ALK (hereafter referred to as the unknown ALK fusion-positive case) (Figure 1) [37]. Two FFPE tissue blocks of ALK-positive tumor cases were also employed, for which the presence of EML4-ALK or KIF5B-ALK had already been confirmed. Total RNA was extracted from each FFPE tissue with the use of the RecoverAll™ Total Nucleic Acid Isolation Kit for FFPE (Applied Biosystems Japan, Tokyo, Japan). The ages of the 3 FFPE blocks used (time from FFPE tissue production to RNA extraction) were 65, 40, and 51 months for the unknown ALK fusion-positive case, EML4-ALK, and KIF5B-ALK, respectively. Written informed consent was obtained from each patient. The study was approved by the institutional review board of the Shizuoka Cancer Center (approval ID 22-J132-22-1) and the Japanese Foundation for Cancer Research (approval ID 2010-1011).

### Modified 5′-RACE for ALK fusions applicable to FFPE tissues

5′-RACE was performed with the SMARTer RACE cDNA Amplification Kit (Clontech) according to the manufacturer's instruction with minor modifications. In brief, instead of the primers included in the kit, ALK-3242R (5′-CTCAGCTTGTACTCAGGGC-3′) was used for cDNA synthesis. The cDNA was subjected to 5′-RACE PCR using PrimeSTAR HS DNA Polymerase (TaKaRa) and the following primers: Universal Primer A Mix of the kit and ALK-3206R (5′-ATGGCTTGCAGCTCCTGGTGCTT-3′). The PCR condition consisted of 5 cycles at 94°C for 30 s and 72°C for 3 min; 5 cycles at 94°C for 30 s, 70°C for 30 s, and 72°C for 3 min; and 30 cycles at 94°C for 30 s, 68°C for 30 s, and 72°C for 3 min.

### FISH

FISH analysis of fusion genes was performed with DNA probes for KLC1 and ALK. Unstained sections (4-µm thick) were subjected to hybridization with an ALK-split probe set (Dako, Tokyo, Japan) or with bacterial artificial chromosome (BAC) clone-derived probes for ALK (RP11-984I21 and RP11-62B19) and KLC1 (RP11-186F6). Hybridized slides were then stained with DAPI and examined using a BX51 fluorescence microscope (Olympus, Tokyo, Japan).

### Synthesis of the putative cDNA of *KLC1-ALK*

**Figure 2:**
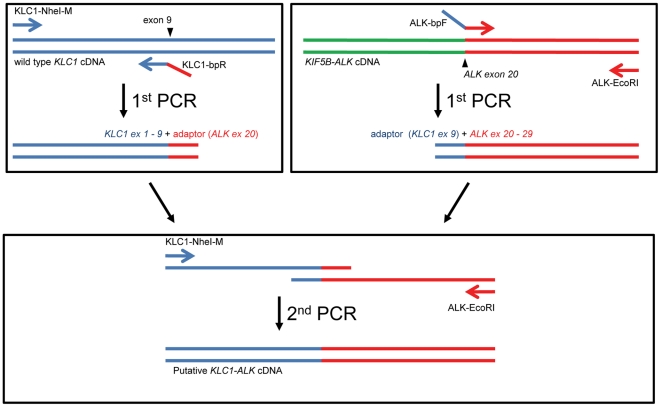
Synthesis of the putative KLC1-ALK full-length cDNA. Two first-round PCRs were performed separately using cDNA synthesized from a tumor tissue expressing KIF5B-ALK with the following primer sets: KLC1-NheI-M and KLC1-bpR, and ALK-bpF and ALK-EcoRI. KLC1-bpR and ALK-bpF had sequences downstream of the ALK break point (exon 20) and upstream of the KLC1 break point (exon 9) as adopter sequences, respectively. Then, the second PCR was performed using a 1/100 dilution of the mixture of the first PCR products as a template with primers KLC1-NheI-M and ALK-EcoRI. The first PCR products were annealed, extended with each other, and then amplified with the primers.

Two independent PCRs were performed using cDNA synthesized from a tumor tissue expressing KIF5B-ALK with the following primer sets: KLC1-NheI-M (5′-GCGCTAGCGAATGTATGACAACATGTCCAC-3′) and KLC1-bpR (5′-GTGCTTCCGGCGGTACACATCTACAGAACCAAACTC-3′), and ALK-bpF (5′-GGGAGTTTGGTTCTGTAGATGTGTACCGCCGGAAGC-3′) and ALK-EcoRI (5′-GATAGAATTCTCAGGGCCCAGGCT-3′). Then, the second PCR was performed using a 1/100 dilution of a mixture of the first PCR products as a template with the KLC1-NheI-M and ALK-EcoRI primers (Figure 2).

### Transformation assay for *KLC1-ALK*

Analysis of the transforming activity of kinase fusions was performed as described previously [9], [38], [39]. A pMXS-based expression plasmid for each fusion was used to generate recombinant ecotropic retroviruses [40], which were then used individually to infect mouse 3T3 fibroblasts. The formation of transformed foci was evaluated after culturing the cells for 4 days. The same set of 3T3 cells was injected subcutaneously into nu/nu mice, and tumor formation was examined after 14 days. The animal experiments were approved by the animal ethics committee of Jichi Medical University (approval ID 1135).

## Results

### Identification of *KLC1-ALK* as a novel *ALK* fusion gene

**Figure 3:**
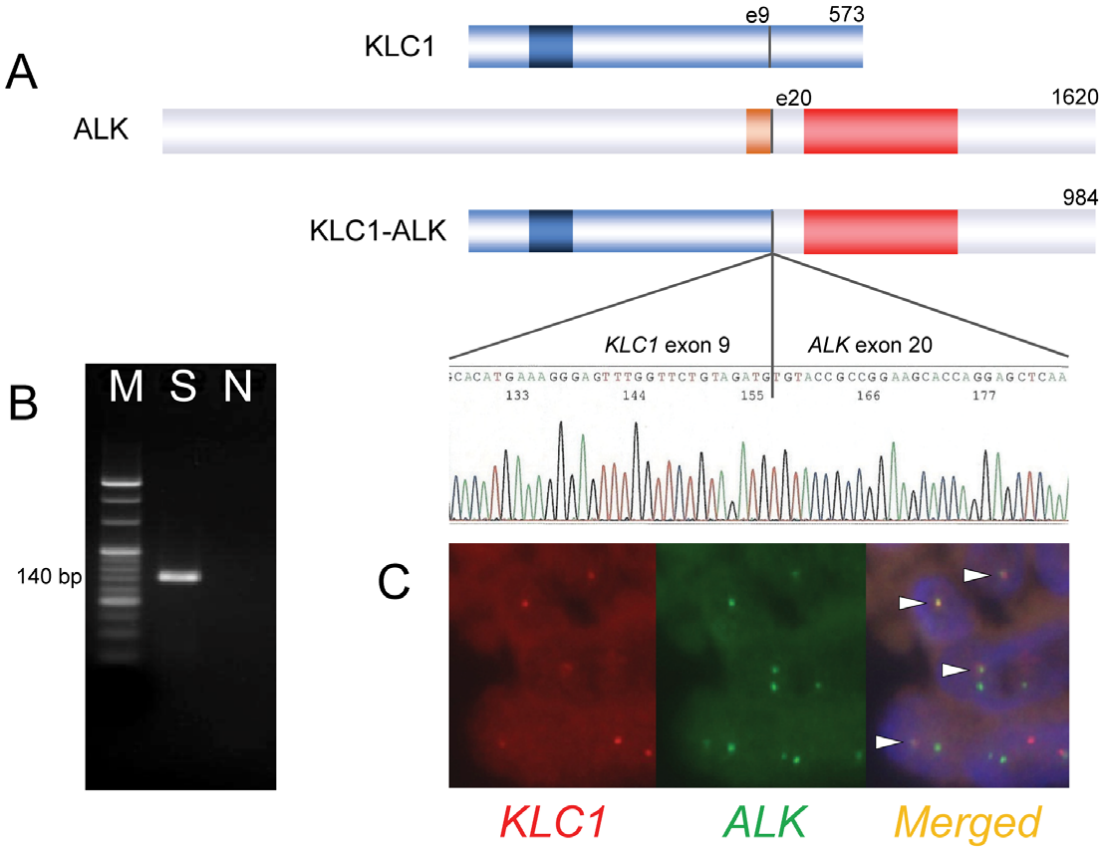
Identification of KLC1-ALK. Panel A shows the schematic structure of KLC1, ALK, and KLC1-ALK proteins and the cDNA sequence around the fusion point. Dark blue, orange, and red parts represent coiled-coil, transmembrane, and kinase domains, respectively. The break point exons and the number of amino acids are indicated. KLC1-ALK-specific RT-PCR using RNA extracted from the FFPE tissue of the unknown ALK fusion-positive case amplified a fragment of the expected product size (140 bp, Panel B) with the consistent fusion sequence (Panel A). A fusion FISH assay for *KLC1-ALK* revealed a fusion signal (yellow) in multiple tumor cells (Panel C). M, marker (100-bp ladder); S, sample (the unknown ALK fusion-positive case); N, no template control.

Our modified 5′-RACE faithfully isolated cDNA fragments for *EML4-ALK* or *KIF5B-ALK* from known ALK- positive tumors (Supplementary Figure S1A and B). We then attempted to isolate cDNA fragments encompassing the fusion points from the unknown ALK fusion-positive case. Nucleotide sequencing of such 5′-RACE products revealed that 2 of 10 clones contained the 3′-terminus of exon 9 of *KLC1* (ENST00000348520) fused to the first nucleotide of exon 20 of *ALK* (ENST00000389048), indicating the presence of a novel fusion between *KLC1* and *ALK*. As this rearrangement constituted an in-frame fusion between the 2 genes, the full-length *KLC1-ALK* cDNA probably produces a protein of 984 amino acids containing an amino-terminal two-thirds of KLC1 and an intracellular region of ALK (Figure 3A). RT-PCR-mediated isolation of a fusion point successfully confirmed the in-frame fusion between the 2 messages (Figure 3A and B). Further, to confirm the genomic rearrangement responsible for the fusion, a fusion FISH assay was performed (Figure 3C). These results were consistent with the presence of t(2;14)(p23;q32.3), leading to the generation of *KLC1-ALK*.

### Transforming potential of *KLC1-ALK*

**Figure 4:**
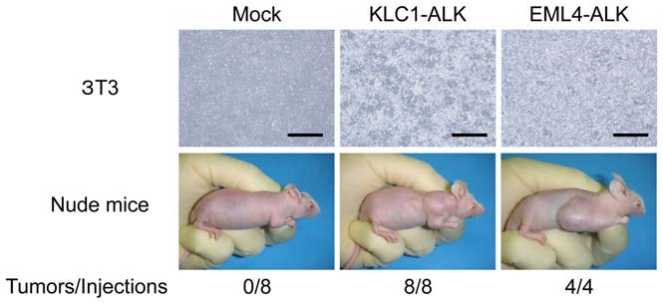
Transforming potential of KLC1-ALK. Upper panels: Mouse 3T3 fibroblasts were infected with retroviruses encoding KLC1-ALK or EML4-ALK or with the corresponding empty virus (Mock). The cells were photographed after 4 days of culture. Scale bar, 1 mm. Lower panels: Nude mice were injected subcutaneously with the corresponding 3T3 cells, and tumor formation was examined after 14 days. The number of tumors formed per injections is indicated at the bottom.

The putative full-length cDNA of *KLC1-ALK* was synthesized from the frozen tissue with KIF5B-ALK fusion expression (Figure 2, Supplementary Figure S2), and was used to generate a recombinant retrovirus expressing the fusion protein with an amino-terminal FLAG epitope tag. Infection of 3T3 cells with the virus expressing KLC1-ALK readily produced multiple transformed foci in culture and subcutaneous tumors in a nude mouse tumorigenicity assay (Figure 4), confirming the potent transforming ability of KLC1-ALK.

## Discussion

Here, by analyzing the FFPE tissues only, we successfully discovered a novel ALK fusion, KLC1-ALK. While snap-frozen materials sampled from biopsied or surgically removed specimens can be used for various types of molecular analyses, they are not routinely sampled in most clinical settings. In contrast, FFPE specimens are usually produced, and histopathology diagnostic archives are an extremely large resource of FFPE tissues in ordinary diagnostic pathology laboratories. However, DNA and RNA extracted from FFPE tissues are severely degraded during formalin fixation and are usually not suitable for assays that need long DNA/RNA of high quality. Recent technical advances have allowed some analyses for known point mutations and known fusion genes, but it is still difficult to identify an unrecognized gene aberration using only an FFPE tissue.

In most *ALK* fusions, the break point of *ALK* is located within intron 19, and the fusion point in mRNA is typically the first nucleotide of exon 20. Therefore, if the primers for 5′-RACE are placed immediately downstream of the first nucleotide of *ALK* exon 20, such 5′-RACE may successfully isolate PCR products containing the partner gene sequence even using FFPE tissues. Based on this hypothesis, we established a 5′-RACE system for *ALK* fusions optimized for FFPE tissues. With this system, we identified a novel *ALK* fusion, *KLC1-ALK*. To the best of our knowledge, this is the first novel oncogenic fusion identified using only an FFPE tissue.

Caution, however, is needed. In some rare cases with *ALK* fusion, the break point of *ALK* fusion mRNA may not be at the 5′-end of exon 20. For example, in variant 4 of *EML4-ALK*, exon 14 of *EML4* is fused to an unknown sequence of 11 bp, which in turn is connected to nucleotide 50 of *ALK* exon 20 (E14;ins11;del49A20) [38]. Our 5′-RACE system would not work on such a case because the reverse primer ALK-3206R corresponds to nucleotides 12–34 of *ALK* exon 20. Therefore, if our modified 5′-RACE fails to isolate fusion cDNAs from cases with a confirmed ALK rearrangement, other primer settings may be attempted.

Kinesin is a heterotetramer of 2 kinesin heavy chains and 2 kinesin light chains, and it moves on the microtubules towards their plus ends carrying various cargos. The heavy chains harbor the motor activity, whereas the light chains play roles in cargo binding and in modulating the activity and subcellular localization of the heavy chains. KLC1 binds to the kinesin heavy chains with an N-terminal domain and to various cargos via the tetratricopeptide repeat domains [41], [42]. Of the 3 histopathologically confirmed ALK fusion partners in lung cancer, EML4 colocalizes with microtubules and may contribute to the stabilization of microtubules [43], KIF5B moves on the microtubules as a kinesin heavy chain [44], and KLC1 binds to kinesin heavy chains as a kinesin light chain. Therefore, it is interesting that all the 3 ALK fusions in lung cancer are likely to colocalize with microtubules.

The most frequent ALK fusion in lung cancer is EML4-ALK (4–7%) [9], [38], and the second is KIF5B-ALK (0.5%) [11]. One case with TFG-ALK is reported [10]. KLC1-ALK may be rare but exists in lung adenocarcinoma, and the patients with this fusion are highly likely to benefit from ALK inhibitor therapy as do patients with other ALK fusions. The incidence may be low, but the significance of this fusion is very high from the perspective of a tailor-made therapeutic option for the patient. Another important point is that KLC1-ALK was found in adenocarcinoma in situ, nonmucinous (formerly called bronchioloalveolar carcinoma, BAC). BAC is recognized to rarely harbor ALK fusions, although a small number of BAC cases has been examined for ALK fusion compared with invasive adenocarcinoma. It would be interesting from a pathobiological perspective to examine a large-scale cohort of BAC and other premalignant conditions for ALK fusion.

There are 3 methods for the detection of ALK fusions: RT-PCR, ALK split FISH, and high-sensitivity anti-ALK immunohistochemistry. For RT-PCR, the 5′ partner gene must be known. Our findings in this study identified one more partner gene that should be targeted in ALK-fusion detection using RT-PCR in lung cancer. The other 2 methods can detect all ALK fusions regardless of fusion partner and, therefore, are suitable for ALK-fusion screening. In other words, these 2 methods cannot identify the fusion partner and need to be succeeded by partner-specific RT-PCR and/or fusion FISH for this purpose. If it is revealed that the partner gene in the tested case is unknown, a novel partner gene is highly likely to be discovered, as was shown in the present study. In fact, using high-sensitivity anti-ALK immunohistochemistry (iAEP method) as screening, we have identified several novel ALK fusions in various types of cancers including lung adenocarcinoma [11], lymphoma [28], sarcoma [35], and renal cell carcinoma [30].

Many efficient tools have been established for the detection of ALK fusion-positive cases using FFPE tissues, including anti-ALK immunohistochemistry and FISH. Our findings will further expand the potential value of archival FFPE tissues and provide further biological and clinical insights into ALK-positive cancers in the forthcoming era of ALK inhibitor therapy.

## Supporting Information

Figure S1 **5′-RACE products using FFPE tissues.** Our modified 5′-RACE faithfully isolated cDNA fragments for *EML4-ALK* (A) or *KIF5B-ALK* (B) from known ALK- positive tumors.
(TIF)

Figure S2 **Putative cDNA sequence of KLC1-ALK.** The putative full-length cDNA of *KLC1-ALK* was synthesized from the frozen tissue with KIF5B-ALK fusion expression.
(PDF)
