# Supplementary figures and images for: KLC1-ALK: A Novel Fusion in Lung Cancer Identified Using a Formalin-Fixed Paraffin-Embedded Tissue Only
Source: PLoS One. 2012 Feb 8;7(2):e31323. doi: 10.1371/journal.pone.0031323 (PMC3275577; doi:10.1371/journal.pone.0031323)

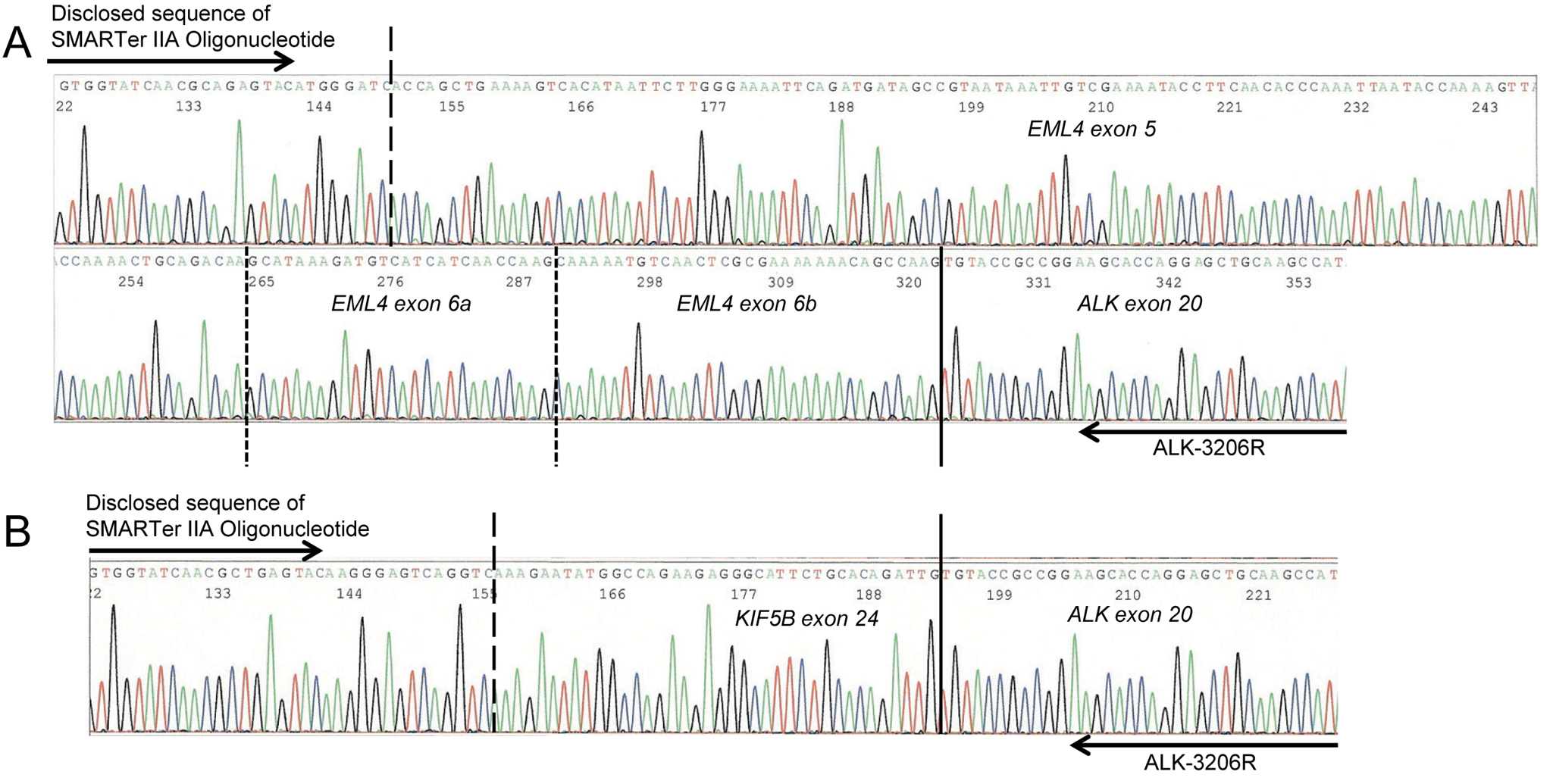

Supplement: Figure S1 — 5′-RACE products using FFPE tissues. Our modified 5′-RACE faithfully isolated cDNA fragments for EML4-ALK (A) or KIF5B-ALK (B) from known ALK- positive tumors. (TIF) [file pone.0031323.s001.tif]
