# Supplementary material for: KLC1-ALK: A Novel Fusion in Lung Cancer Identified Using a Formalin-Fixed Paraffin-Embedded Tissue Only
Source: PLoS One. 2012 Feb 8;7(2):e31323. doi: 10.1371/journal.pone.0031323 (PMC3275577; doi:10.1371/journal.pone.0031323)
Supplement: Figure S2 — Putative cDNA sequence of KLC1-ALK. The putative full-length cDNA of KLC1-ALK was synthesized from the frozen tissue with KIF5B-ALK fusion expression. (PDF) [file pone.0031323.s002.pdf]

Supplementary Figure 2
Putative cDNA sequence of KLC1-ALK

Size   : 2952
Translation Position: 1 -   2952;
Polymorphism: position 830, rs1670283

```
              10        20        30        40        50        60
      ATGTATGACAACATGTCCACAATGGTGTACATAAAGGAAGACAAGTTGGAGAAGCTTACA
  1    M  Y  D  N  M  S  T  M  V  Y  I  K  E  D  K  L  E  K  L  T      20

              70        80        90       100       110       120
      CAGGATGAAATTATTTCTAAGACAAAGCAAGTAATTCAGGGGCTGGAAGCTTTGAAGAAT
 21    Q  D  E  I  I  S  K  T  K  Q  V  I  Q  G  L  E  A  L  K  N      40

             130       140       150       160       170       180
      GAGCACAATTCCATTTTACAAAGTTTGCTGGAGACACTGAAGTGTTTGAAGAAAGATGAT
 41    E  H  N  S  I  L  Q  S  L  L  E  T  L  K  C  L  K  K  D  D      60

             190       200       210       220       230       240
      GAAAGTAATTTGGTGGAGGAGAAATCAAACATGATCCGGAAGTCACTGGAGATGTTGGAG
 61    E  S  N  L  V  E  E  K  S  N  M  I  R  K  S  L  E  M  L  E      80

             250       260       270       280       290       300
      CTCGGCCTGAGTGAGGCACAGGTTATGATGGCTTTGTCAAATCACCTGAATGCTGTGGAG
 81    L  G  L  S  E  A  Q  V  M  M  A  L  S  N  H  L  N  A  V  E     100

             310       320       330       340       350       360
      TCCGAGAAGCAGAAACTGCGTGCGCAGGTTCGTCGTCTGTGCCAGGAGAATCAGTGGCTA
101    S  E  K  Q  K  L  R  A  Q  V  R  R  L  C  Q  E  N  Q  W  L     120

             370       380       390       400       410       420
      CGGGATGAACTGGCCAACACGCAGCAGAAACTGCAGAAGAGTGAGCAGTCTGTGGCTCAA
121    R  D  E  L  A  N  T  Q  Q  K  L  Q  K  S  E  Q  S  V  A  Q     140

             430       440       450       460       470       480
      CTGGAGGAGGAGAAGAAGCATCTGGAGTTTATGAATCAGCTAAAAAAATATGATGACGAC
141    L  E  E  E  K  K  H  L  E  F  M  N  Q  L  K  K  Y  D  D  D     160

             490       500       510       520       530       540
      ATTTCCCCATCCGAGGACAAAGACACTGATTCTACCAAAGAGCCTCTGGATGACCTTTTC
161    I  S  P  S  E  D  K  D  T  D  S  T  K  E  P  L  D  D  L  F     180

             550       560       570       580       590       600
      CCCAATGATGAAGACGACCCAGGGCAAGGAATCCAGCAGCAGCACAGCAGTGCAGCCGCG
181    P  N  D  E  D  D  P  G  Q  G  I  Q  Q  Q  H  S  S  A  A  A     200

             610       620       630       640       650       660
      GCTGCCCAGCAGGGCGGCTACGAGATCCCCGCGCGGCTGCGGACGCTCCACAACCTGGTG
201    A  A  Q  Q  G  G  Y  E  I  P  A  R  L  R  T  L  H  N  L  V     220

             670       680       690       700       710       720
      ATCCAGTACGCCTCGCAGGGGCGCTACGAGGTAGCTGTGCCCCTCTGCAAGCAGGCCCTG
221    I  Q  Y  A  S  Q  G  R  Y  E  V  A  V  P  L  C  K  Q  A  L     240

             730       740       750       760       770       780
      GAGGACCTGGAGAAGACTTCAGGACACGACCACCCGGACGTGGCCACCATGCTCAACATC
241    E  D  L  E  K  T  S  G  H  D  H  P  D  V  A  T  M  L  N  I     260

             790       800       810       820       830       840
      CTGGCCTTGGTGTACAGGGATCAGAATAAATACAAAGATGCAGCTAACCTACTGAATGAT
261    L  A  L  V  Y  R  D  Q  N  K  Y  K  D  A  A  N  L  L  N  D     280
```

```
          850       860       870       880       890       900
     GCCTTGGCTATTCGTGAGAAAACTTTGGGCAAAGATCATCCTGCGGTGGCGGCGACTTTG
281   A  L  A  I  R  E  K  T  L  G  K  D  H  P  A  V  A  A  T  L     300

          910       920       930       940       950       960
     AATAACCTTGCAGTCCTTTATGGTAAAAGAGGGAAGTACAAAGAAGCAGAGCCGTTGTGT
301   N  N  L  A  V  L  Y  G  K  R  G  K  Y  K  E  A  E  P  L  C     320

          970       980       990      1000      1010      1020
     AAAAGAGCTCTGGAAATCCGAGAAAAGGTTTTGGGGAAGGATCACCCCGATGTTGCCAAG
321   K  R  A  L  E  I  R  E  K  V  L  G  K  D  H  P  D  V  A  K     340

         1030      1040      1050      1060      1070      1080
     CAGTTAAATAACTTGGCCTTACTGTGCCAGAACCAGGGCAAGTATGAAGAAGTAGAATAT
341   Q  L  N  N  L  A  L  L  C  Q  N  Q  G  K  Y  E  E  V  E  Y     360

         1090      1100      1110      1120      1130      1140
     TATTATCAAAGAGCCCTCGAGATCTACCAGACAAAACTGGGACCTGATGACCCCAACGTG
361   Y  Y  Q  R  A  L  E  I  Y  Q  T  K  L  G  P  D  D  P  N  V     380

         1150      1160      1170      1180      1190      1200
     GCTAAGACGAAAAATAACCTGGCATCCTGCTATTTGAAACAAGGAAAGTTCAAGCAAGCA
381   A  K  T  K  N  N  L  A  S  C  Y  L  K  Q  G  K  F  K  Q  A     400

         1210      1220      1230      1240      1250      1260
     GAAACACTGTACAAAGAGATTCTCACTCGTGCACATGAAAGGGAGTTTGGTTCTGTAGAT
401   E  T  L  Y  K  E  I  L  T  R  A  H  E  R  E  F  G  S  V  D     420

         1270      1280      1290      1300      1310      1320
     GTGTACCGCCGGAAGCACCAGGAGCTGCAAGCCATGCAGATGGAGCTGCAGAGCCCTGAG
421   V  Y  R  R  K  H  Q  E  L  Q  A  M  Q  M  E  L  Q  S  P  E     440

         1330      1340      1350      1360      1370      1380
     TACAAGCTGAGCAAGCTCCGCACCTCGACCATCATGACCGACTACAACCCCAACTACTGC
441   Y  K  L  S  K  L  R  T  S  T  I  M  T  D  Y  N  P  N  Y  C     460

         1390      1400      1410      1420      1430      1440
     TTTGCTGGCAAGACCTCCTCCATCAGTGACCTGAAGGAGGTGCCGCGGAAAAACATCACC
461   F  A  G  K  T  S  S  I  S  D  L  K  E  V  P  R  K  N  I  T     480

         1450      1460      1470      1480      1490      1500
     CTCATTCGGGGTCTGGGCCATGGCGCCTTTGGGGAGGTGTATGAAGGCCAGGTGTCCGGA
481   L  I  R  G  L  G  H  G  A  F  G  E  V  Y  E  G  Q  V  S  G     500

         1510      1520      1530      1540      1550      1560
     ATGCCCAACGACCCAAGCCCCCTGCAAGTGGCTGTGAAGACGCTGCCTGAAGTGTGCTCT
501   M  P  N  D  P  S  P  L  Q  V  A  V  K  T  L  P  E  V  C  S     520

         1570      1580      1590      1600      1610      1620
     GAACAGGACGAACTGGATTTCCTCATGGAAGCCCTGATCATCAGCAAATTCAACCACCAG
521   E  Q  D  E  L  D  F  L  M  E  A  L  I  I  S  K  F  N  H  Q     540

         1630      1640      1650      1660      1670      1680
     AACATTGTTCGCTGCATTGGGGTGAGCCTGCAATCCCTGCCCCGGTTCATCCTGCTGGAG
541   N  I  V  R  C  I  G  V  S  L  Q  S  L  P  R  F  I  L  L  E     560

         1690      1700      1710      1720      1730      1740
     CTCATGGCGGGGGGAGACCTCAAGTCCTTCCTCCGAGAGACCCGCCCTCGCCCGAGCCAG
561   L  M  A  G  G  D  L  K  S  F  L  R  E  T  R  P  R  P  S  Q     580

         1750      1760      1770      1780      1790      1800
     CCCTCCTCCCTGGCCATGCTGGACCTTCTGCACGTGGCTCGGGACATTGCCTGTGGCTGT
```

```
581   P  S  S  L  A  M  L  D  L  L  H  V  A  R  D  I  A  C  G  C     600

           1810      1820      1830      1840      1850      1860
      CAGTATTTGGAGGAAAACCACTTCATCCACCGAGACATTGCTGCCAGAAACTGCCTCTTG
601   Q  Y  L  E  E  N  H  F  I  H  R  D  I  A  A  R  N  C  L  L     620

           1870      1880      1890      1900      1910      1920
      ACCTGTCCAGGCCCTGGAAGAGTGGCCAAGATTGGAGACTTCGGGATGGCCCGAGACATC
621   T  C  P  G  P  G  R  V  A  K  I  G  D  F  G  M  A  R  D  I     640

           1930      1940      1950      1960      1970      1980
      TACAGGGCGAGCTACTATAGAAAGGGAGGCTGTGCCATGCTGCCAGTTAAGTGGATGCCC
641   Y  R  A  S  Y  Y  R  K  G  G  C  A  M  L  P  V  K  W  M  P     660

           1990      2000      2010      2020      2030      2040
      CCAGAGGCCTTCATGGAAGGAATATTCACTTCTAAAACAGACACATGGTCCTTTGGAGTG
661   P  E  A  F  M  E  G  I  F  T  S  K  T  D  T  W  S  F  G  V     680

           2050      2060      2070      2080      2090      2100
      CTGCTATGGGAAATCTTTTCTCTTGGATATATGCCATACCCCAGCAAAAGCAACCAGGAA
681   L  L  W  E  I  F  S  L  G  Y  M  P  Y  P  S  K  S  N  Q  E     700

           2110      2120      2130      2140      2150      2160
      GTTCTGGAGTTTGTCACCAGTGGAGGCCGGATGGACCCACCCAAGAACTGCCCTGGGCCT
701   V  L  E  F  V  T  S  G  G  R  M  D  P  P  K  N  C  P  G  P     720

           2170      2180      2190      2200      2210      2220
      GTATACCGGATAATGACTCAGTGCTGGCAACATCAGCCTGAAGACAGGCCCAACTTTGCC
721   V  Y  R  I  M  T  Q  C  W  Q  H  Q  P  E  D  R  P  N  F  A     740

           2230      2240      2250      2260      2270      2280
      ATCATTTTGGAGAGGATTGAATACTGCACCCAGGACCCGGATGTAATCAACACCGCTTTG
741   I  I  L  E  R  I  E  Y  C  T  Q  D  P  D  V  I  N  T  A  L     760

           2290      2300      2310      2320      2330      2340
      CCGATAGAATATGGTCCACTTGTGGAAGAGGAAGAGAAAGTGCCTGTGAGGCCCAAGGAC
761   P  I  E  Y  G  P  L  V  E  E  E  E  K  V  P  V  R  P  K  D     780

           2350      2360      2370      2380      2390      2400
      CCTGAGGGGGTTCCTCCTCTCCTGGTCTCTCAACAGGCAAAACGGGAGGAGGAGCGCAGC
781   P  E  G  V  P  P  L  L  V  S  Q  Q  A  K  R  E  E  E  R  S     800

           2410      2420      2430      2440      2450      2460
      CCAGCTGCCCCACCACCTCTGCCTACCACCTCCTCTGGCAAGGCTGCAAAGAAACCCACA
801   P  A  A  P  P  P  L  P  T  T  S  S  G  K  A  A  K  K  P  T     820

           2470      2480      2490      2500      2510      2520
      GCTGCAGAGGTCTCTGTTCGAGTCCCTAGAGGGCCGGCCGTGGAAGGGGGACACGTGAAT
821   A  A  E  V  S  V  R  V  P  R  G  P  A  V  E  G  G  H  V  N     840

           2530      2540      2550      2560      2570      2580
      ATGGCATTCTCTCAGTCCAACCCTCCTTCGGAGTTGCACAAGGTCCACGGATCCAGAAAC
841   M  A  F  S  Q  S  N  P  P  S  E  L  H  K  V  H  G  S  R  N     860

           2590      2600      2610      2620      2630      2640
      AAGCCCACCAGCTTGTGGAACCCAACGTACGGCTCCTGGTTTACAGAGAAACCCACCAAA
861   K  P  T  S  L  W  N  P  T  Y  G  S  W  F  T  E  K  P  T  K     880

           2650      2660      2670      2680      2690      2700
      AAGAATAATCCTATAGCAAAGAAGGAGCCACACGACAGGGGTAACCTGGGGCTGGAGGGA
881   K  N  N  P  I  A  K  K  E  P  H  D  R  G  N  L  G  L  E  G     900

           2710      2720      2730      2740      2750      2760
```

```
      AGCTGTACTGTCCCACCTAACGTTGCAACTGGGAGACTTCCGGGGGCCTCACTGCTCCTA
901    S  C  T  V  P  P  N  V  A  T  G  R  L  P  G  A  S  L  L  L     920

             2770      2780      2790      2800      2810      2820
      GAGCCCTCTTCGCTGACTGCCAATATGAAGGAGGTACCTCTGTTCAGGCTACGTCACTTC
921    E  P  S  S  L  T  A  N  M  K  E  V  P  L  F  R  L  R  H  F     940

             2830      2840      2850      2860      2870      2880
      CCTTGTGGGAATGTCAATTACGGCTACCAGCAACAGGGCTTGCCCTTAGAAGCCGCTACT
941    P  C  G  N  V  N  Y  G  Y  Q  Q  Q  G  L  P  L  E  A  A  T     960

             2890      2900      2910      2920      2930      2940
      GCCCCTGGAGCTGGTCATTACGAGGATACCATTCTGAAAAGCAAGAATAGCATGAACCAG
961    A  P  G  A  G  H  Y  E  D  T  I  L  K  S  K  N  S  M  N  Q     980

             2950
      CCTGGGCCCTGA
981    P  G  P  *     984
```
